# The Role of Molecular and Cellular Aging Pathways on Age-Related Hearing Loss

**DOI:** 10.3390/ijms25179705

**Published:** 2024-09-07

**Authors:** Tuba Ege, Litao Tao, Brian J. North

**Affiliations:** Biomedical Sciences Department, School of Medicine, Creighton University, Omaha, NE 68178, USA; tubaege@creighton.edu

**Keywords:** hearing loss, cochlear aging, hair cells, senescence, sirtuins, mitochondrial dysfunction

## Abstract

Aging, a complex process marked by molecular and cellular changes, inevitably influences tissue and organ homeostasis and leads to an increased onset or progression of many chronic diseases and conditions, one of which is age-related hearing loss (ARHL). ARHL, known as presbycusis, is characterized by the gradual and irreversible decline in auditory sensitivity, accompanied by the loss of auditory sensory cells and neurons, and the decline in auditory processing abilities associated with aging. The extended human lifespan achieved by modern medicine simultaneously exposes a rising prevalence of age-related conditions, with ARHL being one of the most significant. While our understanding of the molecular basis for aging has increased over the past three decades, a further understanding of the interrelationship between the key pathways controlling the aging process and the development of ARHL is needed to identify novel targets for the treatment of AHRL. The dysregulation of molecular pathways (AMPK, mTOR, insulin/IGF-1, and sirtuins) and cellular pathways (senescence, autophagy, and oxidative stress) have been shown to contribute to ARHL. However, the mechanistic basis for these pathways in the initiation and progression of ARHL needs to be clarified. Therefore, understanding how longevity pathways are associated with ARHL will directly influence the development of therapeutic strategies to treat or prevent ARHL. This review explores our current understanding of the molecular and cellular mechanisms of aging and hearing loss and their potential to provide new approaches for early diagnosis, prevention, and treatment of ARHL.

## 1. Introduction

As a fundamental aspect of human biology, aging is a twisted biological continuum characterized by a physiological decline stemming from progressive dysregulation in a range of molecular and cellular processes [1], which plays a significant role in the onset of numerous chronic diseases like heart disease, kidney disease, and cancer; these conditions impact both healthspan and lifespan and contribute to increased mortality rates [2]. The rate and intensity of changes in molecular and cellular aging pathways differentiate healthy aging from pathological aging and disease progression.

Age-related hearing loss (ARHL), recognized as the third most common chronic geriatric disease [3], affects approximately half of adults aged 85 years and over [4], significantly impairing the health and well-being of the elderly population, leading to communication challenges, social isolation, and cognitive decline [5]. The relationship between aging and ARHL is complex, as the same molecular and cellular mechanisms that drive the aging process also contribute to the deterioration of auditory function. As the body ages, the auditory system becomes increasingly susceptible to the cumulative effects of multiple degenerative processes associated with aging, leading to the progressive hearing loss characteristic of ARHL [6]. Despite advancements in identifying the age-related cellular and molecular changes in the inner ear, the long-standing question that remains is which precise mechanisms underlie the age-dependent degeneration of cochlear structure and function, as well as which methods can be used to preserve or reverse these processes.

Dysregulation of cellular pathways like senescence, autophagy, and oxidative stress, in addition to molecular pathways regulated by AMP-activated protein kinase (AMPK), the mechanistic targets of rapamycin (mTOR), insulin/insulin-like growth factor-1 (IGF-1), and sirtuins (SIRTs) have each been implicated in hearing loss progression, but the specific causative factors and their direct roles on molecular and cellular pathways that lead to cochlear degeneration are not fully elucidated. Understanding how these pathways affect postmitotic hair cells, the stria vascularis, and the spiral ganglion cells is vital for elucidating the mechanisms of ARHL and developing therapeutic interventions to prevent or mitigate ARHL. Calorie restriction (CR), well recognized for its healthspan and lifespan-extending properties [7,8], has also been shown to slow ARHL in both rodents and primates [9,10], but the specific molecular pathways modified by CR in the inner ear and the most effective CR mimetic compounds remain unclear. However, molecules targeting oxidative stress and mitochondrial dysfunction or using CR mimetics such as metformin and nicotinamide mononucleotide (NMN), as well as the potential of senolytics or senomorphics, may offer new treatment strategies for ARHL. Characterizing these fundamental aging pathways will not only enhance our understanding of general aging processes but also illuminate their role in ARHL. In this review, we first discuss our current knowledge of aging and ARHL pathways and how small molecule agents modulate these pathways. Later, we focus on how targeting these pathways may provide therapeutic interventions for ARHL prevention or treatment.

## 2. Aging and Hearing Loss: Integrated Pathways

As a natural and complex biological process, aging is a significant risk factor for the progression of chronic diseases, including ARHL [11]. It is characterized by the disruption of tissue homeostasis, altered physiological functions, and a decreased capability to overcome external stresses. These changes lead to increased incidence of diseases and mortality [12]. Throughout life, exposure to both external and internal damaging factors, coupled with the diminishing capacity for repair and maintenance, reduces cellular and tissue resilience, ultimately resulting in their inability to maintain homeostasis [13].

Hearing loss, the fourth leading cause of disability worldwide [14], can occur at any age [6] and is influenced by genetic mutations, noise exposure, therapeutic drug exposure, smoking, adiposity, diabetes, and aging [15]. The peripheral auditory system, comprising the external, middle, and inner ear with the cochlear nerve, converts sound wave pressure into neural signals transmitted to the central nervous system [16].

The inner ear consists of the cochlea and semicircular canals, playing distinct roles in hearing and balance, respectively. In the cochlea, vibrations from the oval window are transmitted first to the perilymph and then to the endolymph in the cochlear duct [17]. The organ of Corti, resting on the basilar membrane within the cochlea, contains one row of medial inner hair cells (IHCs) and three rows of lateral outer hair cells (OHCs), together with the supporting cells [18,19]. These cells are connected to the spiral ganglion neurons (SGNs), which relay auditory signals to the brain stem via the auditory nerve [17]. When moved by sound-induced vibrations, the hair cells’ (HCs) stereocilia open ion channels, leading to cell depolarization due to K^+^ influx [20]. This depolarization prompts IHCs to release neurotransmitters, initiating signal transmission along the auditory pathway [21,22]. Defects in any of these processes can result in hearing loss. Notably, HCs are susceptible to damage from ototoxic drugs and oxidative stress. Once lost, these cells cannot regenerate in mammals, and their loss can lead to spiral ganglion degeneration [22]. 

ARHL is the most common sensory deficit in the elderly population. It is characterized by a progressive, typically bilateral decline in auditory function and temporal processing [23], involving the degeneration of the cochlea and loss of auditory nerve fibers [24]. It emerges as a significant geriatric health problem, afflicting approximately one-third of the elderly population worldwide [25,26]. This condition not only adversely affects older individuals by impairing communication [27] but also contributes to social isolation [28], depression [29], and a reduction in physical and cognitive functions [30]. Moreover, hearing impairment has been linked to senile dementia [28]. While aging is the primary risk factor for ARHL, other contributors include genetic predisposition, sex, diet, socioeconomic factors, noise exposure, ototoxic medications, and health comorbidities [31]. 

The pathology of ARHL encompasses many components, including the sensory hair cells, stria vascularis (which maintains the ionic composition of endolymph), spiral ligament, and afferent SGNs, as well as the central auditory pathways [28]. Degeneration in the spiral ligament and the stria vascularis, coupled with a decrease in ATPase expression, impairs the endocochlear potential generated by the stria vascularis. This reduction in endocochlear potential negatively impacts cochlear amplification, primarily caused by the electromotility of the OHCs, leading to high-frequency hearing loss [32]. Similarly, the degeneration of the sensory cells, especially the OHCs, and their electromotility contributes to hearing loss [33]. Furthermore, neuronal degeneration impairs sound discrimination [34], indicating that the pathology of ARHL may arise from mitochondrial dysfunction, increased ROS production, and disturbances in cellular and/or molecular pathways related to aging. A comprehensive study using CBA/J mice, a model for human ARHL, provided deeper insights into the molecular and cellular changes in cochlear HCs associated with aging. Auditory brainstem response (ABR) and distortion product otoacoustic emissions (DPOAE) thresholds increased from 18 months, progressively worsening over time. HC loss, more severe in the OHCs than the IHCs, follows a base-to-apex gradient, accompanied by morphological changes like stereocilia elongation and fusion. Recent studies, including RNA-seq analysis of aged hair cells isolated from mice, have provided insights into the molecular changes associated with aging in cochlear HCs. This study revealed the transcriptional alterations that affect various cellular and molecular pathways, indicating shifts from pro-survival to pro-apoptotic and pro-inflammatory pathways, which enhance the progression of ARHL. Such shifts contribute to the degeneration observed in sensory cells, particularly OHCs, and their electromotility, highlighting the complexity of ARHL’s pathophysiology [35].

The progression of aging is governed by multiple biological and molecular mechanisms, including telomere attrition, mitochondrial dysfunction, genomic instability, epigenetic alterations, loss of proteostasis, cellular senescence, stem cell depletion, deregulated nutrient sensing, and abnormal intercellular communication [36,37,38]. At the molecular level, these hallmarks often involve key regulators such as mTOR, IGF-1, AMPK, SIRTs, p66^shc^, and BubR1 [1,39,40]. While molecular and cellular pathways are strongly linked to both aging and auditory function in ARHL, it is essential to recognize that additional factors may also play a role in ARHL. Although our focus is on the well-established mechanisms of aging and their consequences in the inner ear, the complex nature of ARHL involves multiple biological processes, and understanding these processes helps us explore the potential therapeutic interventions for ARHL prevention or treatment.

### 2.1. Cellular Aging and Its Impact on Hearing

**Figure 1:**
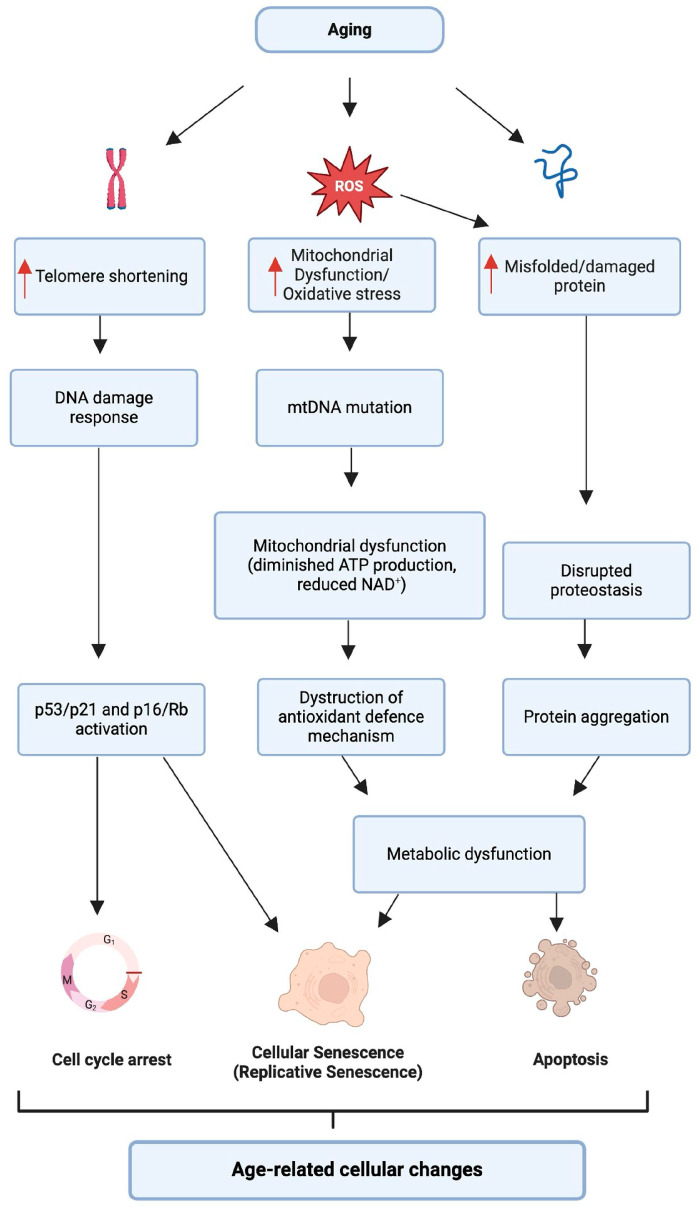
Diagram of interconnected molecular pathways describing biological processes associated with aging. As we age, several vital processes are disrupted: increased reactive oxygen species (ROS) lead to mitochondrial damage and oxidative stress; telomeres shorten, leading to a chronic DNA damage response; proteins misfold, DNA is damaged, and mitochondrial DNA mutations occur. These changes activate cell cycle inhibitors, leading to cellular senescence or apoptosis, and impair the proteostasis network, resulting in protein aggregation. These cellular events cause metabolic imbalance and contribute to age-related cellular changes, which can manifest in various age-related conditions, including hearing loss.

The onset of various age-related diseases, including ARHL, is often attributed to the dysregulation of fundamental cellular mechanisms, such as telomere attrition leading to genomic instability, oxidative stress contributing to cellular damage, impaired autophagy affecting cellular homeostasis, disrupted proteostasis impacting protein folding, and senescence affecting cell renewal and turnover [41]. These pathways are crucial mechanisms for maintaining healthy aging and, when disrupted, they contribute to the cellular degeneration observed in ARHL, as illustrated in Figure 1. The accumulated cellular damage impairs the function of auditory cells, leading to the hearing loss characteristics of ARHL [42].

#### 2.1.1. Telomere Attrition

Telomeres, the repetitive DNA sequence at the ends of linear chromosomes, protect genomic integrity [43]. In the 1980s, the discovery of telomeres in the single-celled organism *Tetrahymena* [44] led to the identification of telomerase, a ribonucleoprotein that synthesizes telomeres [45]. While telomerase is expressed in stem cells and cancer cells, it has low or undetectable levels in human somatic cells, including those in the inner ear [46]. Telomere shortening elicits a persistent DNA damage response promoting the activation of the p53–p21 and p16–pRB pathways, leading to cell cycle arrest and cellular senescence (replicative senescence) [47,48]. Although inner ear cells are primarily non-dividing, telomere attrition can still impact their function through increased DNA damage response and altered gene expression, potentially contributing to age-related cellular dysfunction [49].

Recent studies have shown that the variants of the telomere maintenance gene including *RTEL1* may be associated with ARHL in humans [50]. Animal studies provide more direct evidence as mice with short telomeres or telomerase deficiency exhibited accelerated hearing loss and cochlear degeneration [51]. These mouse models mimic accelerated human aging, suggesting that telomere attrition contributes to ARHL pathogenesis, potentially through increased cellular senescence and apoptosis in cochlear cells. 

While a correlation between longer telomeres and better health is established in centenarians [52,53], the specific relationship between telomere shortening and ARHL requires further investigation. Future research should consider that hair cells and supporting cells in mammalian cochleae are postmitotic, making it unlikely that telomere attrition through DNA replication directly contributes to ARHL in these cell types. Studies should focus on how telomere attrition affects other cochlear cell types and whether telomere-targeted interventions could prevent or delay ARHL. Preliminary studies in other age-related conditions have shown promise for such interventions [54], but their efficacy in ARHL prevention remains to be determined. 

#### 2.1.2. Oxidative Stress

Mitochondria serve as the primary source of cellular energy through oxidative phosphorylation and are involved in apoptosis and signal transduction [55]. A byproduct of mitochondrial oxidative respiration is the generation of reactive oxygen species (ROS), which can damage cellular macromolecules [56], including the mitochondrial genome (mtDNA). This can disrupt respiratory complex efficiency and enhance further ROS production in a vicious cycle.

ROS can be removed by antioxidant enzymes, including superoxide dismutase, catalase, and glutathione peroxidase [57]. Targeting catalase to the mitochondria to reduce oxidative stress has been shown to extend the lifespan of mice [58,59]. While the reactive oxygen theory of aging is still debated, it is clear that ROS contributes to cellular dysfunction, cellular senescence, mitochondrial impairment, and inflammation, making oxidative stress management crucial for mitigating aging and ARHL [60]. 

ROS plays a prominent role in cochlear aging through mitochondrial dysfunction [61]. Lipid peroxidation, oxidative mitochondrial DNA damage, and glutathione-conjugated proteins are markers of cochlear aging in mice [62]. The expression levels of antioxidant enzymes such as catalase, Sod1, and Sod2 decrease in aged mice. Loss of cytosolic Sod1 leads to increased ARHL, reduced thickness of the stria vascularis, and degeneration of the spiral ganglion neurons [62]. The absence of antioxidant enzymes Gpx1 or Sod1 elevates susceptibility to noise-induced hearing loss, whereas catalase overexpression mitigates hair cell loss [63]. Additionally, low levels of melatonin, a ROS scavenger, are linked to hearing loss in the elderly [62]. Lipofuscin, known as the aging pigment, accumulates in cells as a marker of aging; its buildup in the SG neurons’ cytoplasm in ARHL cases signifies diminished cellular efficiency [24]. 

ARHL is thought to involve ROS-induced, BAK-mediated apoptosis, a process relevant to the aging of multiple tissues like the brain and cochlea [15]. Accumulation of mitochondrial mutations leading to mitochondrial dysfunction and altered energy metabolism disrupts cochlear function. Cochlear cell sensitivity to energy metabolism changes can trigger apoptosis, causing neuron and hair cell death [61]. BAK and BAX, pro-apoptotic proteins of the Bcl-2 family, facilitate mitochondrial-mediated apoptosis by forming pores in the outer mitochondrial membrane, leading to the release of cytochrome c and other pro-apoptotic factors [64]. Bak deletion protects mice from ARHL and enhances the survival of cochlear neurons and outer hair cell survival [65]. p53 is suggested to play a key role in this process, with mitochondria-derived ROS in the aged cochlea causing oxidative DNA damage and leading to activation of p53. Consequently, p53 activates Bak on the mitochondrial outer membrane to induce apoptosis [64]. *Bak^−/−^* mice exhibited reduced cochlear cell loss and preserved hearing, suggesting that inhibiting Bak could prevent ARHL [61]. 

In ARHL, an imbalance of ROS impairs antioxidant function, leading to the premature onset of hearing loss, as evidenced in SAMP8 mice, which exhibited early hearing loss and the sequential degeneration of cochlear components [42]. In vitro studies using the HEI-OC1 auditory cell line have corroborated these findings. When exposed to hydrogen peroxide to mimic oxidative stress, these cells exhibit characteristics of senescence, including reduced viability and an increased expression of senescence markers, providing a valuable model for studying ROS-induced damage in ARHL at the cellular level [66]. Aging-related impairment in mitophagy, leading to the buildup of damaged mitochondria and loss of mitochondrial integrity, leads to an increase in ROS levels and contributes to significant changes in the central auditory system including increased ABR thresholds in C57BL/6J mice [67]. Oxidative stress also induces cellular changes and triggers an inflammatory response in both the peripheral cochlea and the central auditory system in CBA/CaJ mice [68], highlighting a critical role for ROS in ARHL progression.

#### 2.1.3. Proteostasis

Protein homeostasis, or proteostasis, is essential for maintaining the proteome and regulating aging [69]. This balance involving protein synthesis, folding, maintenance, degradation, and organelle turnover, is controlled by chaperones, the ubiquitin–proteasome system (UPS), and the autophagy–lysosomal system [70,71]. Chaperones, in the form of heat shock proteins (HSPs), decide the refolding or degradation of unstable proteins [72], a process regulated by the heat shock response (HSR) through heat shock transcription factors (HSFs) [73]. In the auditory system, maintaining proteostasis is particularly crucial due to the high metabolic demands of cochlear hair cells and their limited regenerative capacity.

The heat shock response declines with age, possibly through declining HSF1 protein levels as well as its activation and DNA-binding capacity [74]. Age-related elevations in the endoplasmic reticulum (ER) stress and reduction in cellular ATP bioavailability and chaperone function exacerbate the decline in protein maintenance [75], leading to the accumulation of harmful protein modifications such as glycation [76]. Misfolded or damaged proteins are degraded by the ubiquitin–proteasome pathway or through lysosome-mediated autophagy [77]. Age-related reductions in ubiquitination [78] and proteasome activity contribute to the aggregation of misfolded proteins, characteristic of diseases like Alzheimer’s and Parkinson’s [79], showing the importance of proteostasis in aging and its potential role in ARHL.

Enhancing HSF1 through overexpression has shown significant protective effects against ARHL, particularly in the high-frequency region. This is achieved by a reduction in ER stress markers (CHOP), a decline in apoptosis-related proteins (cleaved caspase-3), and an elevation in HSP40 and HSP70 proteins [75,80]. These findings suggest that pharmacological interventions targeting the upregulation of HSPs could effectively alleviate ARHL. The protective effect of HSPs may be due to their role in preventing protein aggregation and maintaining the function of key proteins in auditory hair cells and neurons. Additionally, the ubiquitin–proteasome pathway emerges as another key player in modulating proteins associated with aging and cellular stress responses, hinting at its involvement in the auditory system’s age-related degeneration [81]. Impaired proteasome function in cochlear cells could lead to the accumulation of damaged proteins, potentially contributing to cellular dysfunction and death in ARHL.

#### 2.1.4. Autophagy

Autophagy is a crucial process for maintaining homeostasis and protecting against stress by breaking down misfolded or aggregated proteins and eliminating damaged organelles [82,83]. There are the following three distinct types of autophagy: microautophagy, chaperone-mediated autophagy (CMA), and macroautophagy, each with unique characteristics and functions [84]. Microautophagy involves the direct engulfment of cytoplasmic elements [85], while CMA targets specific proteins for lysosomal degradation [86], and macroautophagy forms autophagosomes to engulf cellular elements [87]. Aging reduces lysosomal protease activity, key autophagy regulators such as LC3 and ATG7, and transcription of autophagy genes, resulting in a decline in autophagic capacity [88]. The compensatory relationship between macroautophagy and CMA also collapses with age [76], further linking this decline to age-related diseases [89]. The age-related decline in autophagy efficiency correlates with increased cellular damage in various tissues, including auditory structures.

Autophagy plays an essential role in developing and preserving HCs; for instance, mice lacking Atg5 exhibit severe congenital hearing loss and HC degeneration [90]. Autophagy supports the survival of HCs and spiral ganglion neurons by mitigating stress-induced damage and maintaining cellular homeostasis [91]. In vitro studies using cochlear HC cultures have shown that pharmacological enhancement of autophagy can protect against ototoxic drug-induced cell death, offering potential therapeutic strategies for ARHL [92]. Enhanced autophagy, either through moderate noise exposure or pharmacological means, can protect HCs from death [93], suggesting that autophagy regulation holds critical implications for preventing ARHL. However, dysregulated autophagy can contribute to HC death, exacerbating hearing loss [94], making it a potential therapeutic target for age-related disorders and ARHL.

Interestingly, centenarians exhibit elevated expression of autophagy genes (CTSB, ATP6V0C, ATG4D, and WIPI1), implying a better preserved autophagic mechanism [95] that has contributed to more effective cellular homeostasis and longevity [96]. This preservation of autophagy likely plays a role in maintaining hearing function and delaying the onset of age-related auditory decline, offering insights into potential interventions for ARHL. 

#### 2.1.5. Cellular Senescence

Cellular senescence has been linked to cellular aging [46,97] and significantly impacts tissue homeostasis and organismal aging [98]. While traditionally viewed as irreversible [99], recent studies suggest that cellular senescence may be reversible under certain conditions [100]. Senescent cells are characterized by altered gene expression profiles, increased resistance to apoptosis, and enhanced protein synthesis [101]. They show specific markers, including increased cell size and intracellular protein content, heightened senescence-associated β-galactosidase (SA-β-gal) activity, persistent DNA damage foci, and the SASP (senescence-associated secretory phenotype) [102], which are regulated by the mTOR and AMPK pathways [103,104]. The SASP, composed of pro-inflammatory factors, plays a critical role in sustaining inflammatory stress, which is closely associated with aging and ARHL [105].

Senescence is triggered by various factors, such as telomere attrition, ROS accumulation, persistent DNA damage, and oncogene activation [106,107,108], leading to the activation of cell cycle inhibitors including p16/Rb and p53/p21^CIP^ [109]. In aging cochlear cells, oxidative stress and inflammation are important, contributing to ARHL [42]. ROS-induced DNA damage in these cells triggers p53 signaling, elevating p21 levels and promoting senescence [62], marked by increased p38 phosphorylation. While ROS-induced DNA damage does not significantly affect p16 levels, it notably reduces the expression of BubR1 and p19. Reduced p19 may alleviate senescence-like symptoms in cochlear cells through regulating BubR1 [62,110], although the role of BubR1 in hearing loss remains unexplored. SAMP8 mice, known for premature senescence similar to BubR1 hypomorphic animals, exhibit decreased Nrf2 and Sod2 levels and elevated p66^Shc^, which mediates cellular oxidative stress levels [111] and the Ras/MAPK kinase cascade [112], leading to increased ROS, apoptosis, and consequently hair cell damage and hearing loss [111]. Oxidative stress activates PKCβ, which phosphorylates serine-36 of p66^Shc^, triggering further ROS production and downregulating antioxidant enzymes such as glutathione peroxidase-1 via FOXO3a [111]. Inhibition of p66^Shc^ expression is known to extend the lifespan of mice by resisting oxidative stress and may also confer benefits to the marginal cells in the stria vascularis, indicating a potential strategy for mitigating oxidative stress in these auditory cells [111]. Moreover, increased autophagy and impaired autophagic flux have been linked to diminished hearing capacities in SAMP8 mice. Together, these studies indicate that suppressing ROS-induced senescence may play an important role in maintaining adult hearing, especially under auditory stress or aging [62]. 

Mitophagy, the removal of dysfunctional mitochondria, is essential for cell survival, and cochlear function is also linked to senescence. BNIP3L/NIX, which facilitates mitophagy in auditory cells, shows a protective role against ARHL. Mice lacking BNIP3L/NIX show reduced mitophagy and increased sensitivity to oxidative stress, leading to heightened cellular senescence [113]. Conversely, overexpressing BNIP3L/NIX in mice accelerates the degradation of TOMM20 and LC3B, highlighting its protective role [113]. Additionally, altered dynamin-related protein-1 (DRP-1) expression disrupts mitophagy in the aged cochlea, driving hair cells to senescence and exacerbating ARHL [114]. Thus, suppressing ROS-induced senescence and promoting mitophagy is essential for maintaining hearing, especially under cellular stress or aging. These findings show the essential role of cellular senescence in the progression of ARHL, highlighting its potential for therapeutic intervention targeting senescence pathways.

### 2.2. Molecular Aging Pathways and Their Role in Hearing Loss

**Figure 2:**
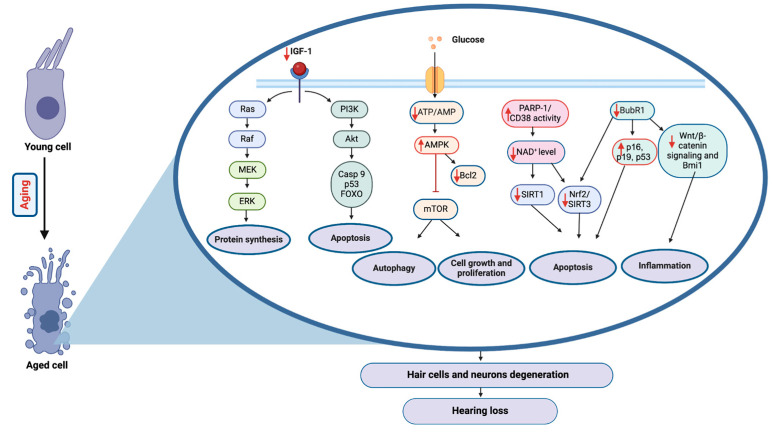
Overview of the molecular pathways involved in aging and hearing loss. IGF-1 and glucose metabolism pathways intersect with cellular processes. Aging is associated with reduced IGF-1 signaling, affecting the Ras/Raf/MEK/ERK cascade critical for protein synthesis and cell survival. Decreased glucose availability alters the ATP/AMP ratio, leading to increased AMPK activity and subsequent effects on the mTOR pathway, which is essential for cell growth and proliferation. Moreover, age-related decline in NAD^+^ level impacts SIRT1 activity, a key regulator of apoptosis and inflammation, through its action on the Nrf2/SIRT3 pathway. These pathways and alterations in Bcl2, Caspase 3/9, FOXO, and cell cycle regulators such as p16, p19, and p53 lead to increased cellular apoptosis and autophagy. Concurrently, dysregulation of the Wnt/β-catenin signaling and inflammation through BubR1 and Bmi1 contribute to this process. The culmination of these molecular events results in the degeneration of hair cells and neurons in the auditory system, leading to hearing loss.

Molecular regulators of aging have been identified through studies in various model organisms, including *Saccharomyces cerevisiae*, *Caenorhabditis elegans*, *Drosophila melanogaster*, and mice. Among these, the most highly conserved regulators include mTOR, IGF-1, AMPK, and Sirtuins. Additionally, BubR1 has been recently recognized as playing a significant role in regulating aging in mice. These molecular pathways collectively influence tissues and organ function over time, contributing to the aging process and increasing susceptibility to chronic diseases [115]. Although the cellular changes associated with ARHL are better understood, its molecular mechanism remains ambiguous. However, studies have defined the influence of the aging pathways described above on the development of ARHL, as illustrated in Figure 2.

#### 2.2.1. Insulin/IGF-1 Pathway

The insulin/IGF-1 signaling pathway (IIS) is crucial for regulating aging and controlling protein synthesis, glucose metabolism, and cellular proliferation and differentiation, while also managing oxidative stress and inflammation. Its significance in lifespan regulation was first discovered in *C. elegans*, where the deletion of the insulin/IGF-1 receptor homolog daf-2 doubled the lifespan via activating the transcription factor DAF-16 [116]. Similar longevity was observed in the Snell, Ames, and Laron dwarf mice, all showing reduced insulin-like growth factor levels and thus suggesting a conserved mechanism [117,118,119]. 

IGF-1, crucial during development, decreases with age but remains active [120], initially enhancing growth but, over time, it contributes to aging by activating certain signaling pathways, including the FOXO, NF-κB [121], MAPK, and the PI3K/Akt pathways [122]. IGF-1 also upregulates the Nrf2/Sirt3 pathway, stimulates mitophagy [123], and activates inflammatory responses through PI3K/Akt [124]. However, under certain conditions, Insulin/IGF-1 may suppress FOXO transcription factors [125], possibly inhibiting NF-κB signaling through FOXOs and SIRT1 [126], and downregulate cyclin-dependent kinase inhibitors like p16 and p21 thus preventing senescence [126]. 

IGF-1 serves as a neuroprotective factor [127] crucial for the maintenance of cellular metabolism in the inner ear [128,129]. It regulates the development and maintenance of cochlear hair cells, supports the regeneration of cochlear synapses, and aids synaptic neurotransmission in the cochlear nuclei [128,130]. IGF-1 signaling may protect cochlear cells from apoptosis [131] through the MAPK and Akt pathways, potentially shielding against ARHL. It promotes the survival and regeneration of cochlear hair cells, emphasizing its crucial role in auditory function [132].

While reduced IGF-1 signaling is associated with an increased lifespan, IGF-1 deficiency in animals leads to sensorineural hearing loss [129,133], increased inflammation, and loss of cell renewal mechanisms, adversely affecting the cochlea and vestibular organs, essential for hearing and balance [129]. *Igf-1^−/−^* mice exhibit cochlear neuronal loss, profound deafness from the onset of hearing, and premature degeneration of the stria vascularis [120]. In humans, decreased IGF-1 levels with aging correlate with worsening hearing issues. Overall, the relationship between the GH/insulin/IGF-1 signaling pathway and aging in humans is more nuanced than in model organisms, highlighting the multifaceted role of IGF-1 in aging and anti-aging processes. This complexity underscores the need for careful consideration when targeting the IGF-1 pathway for ARHL prevention or treatment.

#### 2.2.2. mTOR Pathway

The mTOR pathway is a crucial negative regulator of lifespan, controlling cell growth, metabolism, and autophagy across species [134]. This conserved Ser/Thr protein kinase balances cellular anabolism and catabolism. When activated, mTOR Complex 1 (mTORC1) promotes protein, nucleotide, lipid synthesis, and mitochondrial biogenesis while suppressing autophagy in response to growth factors, nutrients, and increased energy levels [135]. mTORC1 activity is enhanced by PI3K/Akt and Ras–Raf–MEK–ERK signaling cascades, while factors like Wnt signaling, hypoxia, DNA damage, and cell stress inhibit it [136]. Reduced PI3K/AKT/mTOR signaling extends the lifespan of various organisms [137], whereas its sustained activation is associated with cognitive decline and Alzheimer’s disease [138].

The mTOR pathway influences the physiology of inner hair cells and the progression of ARHL. Age-related mitochondrial dysfunction elevates ROS levels, promoting cellular senescence and increasing peroxisome numbers [111], which play a role in the regulation of mTORC1 signaling in the inner hair cells. The mTORC1 complex, consisting of mTOR, Raptor, and mLST8 [139], is negatively regulated by TSC1 and TSC2 and is located on the peroxisome membranes [140]. Hyperactivation of mTORC1, mediated by TSC inactivation, promotes ARHL [140]. Deletion of TSC1 in mice hyper-activates mTORC1 signaling, inducing oxidative stress and impairing autophagy, leading to damaged hair cells and hearing loss. Conversely, the deletion of Raptor, which inactivates mTORC1 signaling, delays the onset of ARHL. Elevated phosphorylation of S6, a target of mTORC1, in cochlear hair cells of aged mice indicates increased mTORC1 activity with age [140]. S6 phosphorylation is a key indicator of mTORC1 activity, and its elevation is associated with enhanced protein synthesis and cellular growth. These findings suggest a potential link between mTORC1 activity and the progression of ARHL. The observed delay in ARHL onset with mTORC1 inhibition through Raptor deletion suggests a potential area for further research into the role of mTOR pathway modulation in ARHL progression [140].

#### 2.2.3. AMPK Pathway

AMPK, a conserved serine/threonine kinase, regulates several pathways linked with energy balance and longevity, with its dysregulation leading to chronic diseases [141]. It plays a crucial role in caloric restriction, extending lifespan by increasing NAD^+^ levels and stimulating SIRT1 [142]. AMPK reduces ATP consumption and enhances the catabolic pathways during nutrient scarcity [143,144], exhibiting antagonistic effects on mTOR to influence autophagy and protein synthesis [145]. It inhibits mTORC1 and protein synthesis [146], promoting autophagy by phosphorylating ULK1 and Atg13, while mTOR suppresses these same processes at different regulatory sites. Autophagy and B cell lymphoma 2 (BCL2) protein inhibits apoptosis and delay senescence [147]. Activation of AMPK, influenced by mitochondrial ROS, supports longevity through a PGC-1α-dependent antioxidant response [142].

Autophagy, regulated by AMPK and mTOR signaling, experiences a decline with age in the auditory cortex [148]. The accumulation of ROS activates AMPK, which promotes autophagy by phosphorylating and activating early autophagy initiation factors [148]. However, deficiencies in autophagy and the AMPK–mTOR–ULK1 signaling pathway limit the protective function of autophagy, leading to degeneration of the auditory cortex via accelerated apoptosis and senescence [148]. Interestingly, AMPK activation has been linked to accelerated hearing loss in Tg-mtTFB1 mice, a mitochondrial deafness mouse model [149]. In AMPKα1 depleted mice, a reduction in pro-apoptotic signaling (Bax) and an increase in anti-apoptotic (Bcl-2) signaling were observed within the cochlea. Thus, the downregulation of AMPK may prevent apoptosis via a ROS–AMPK–Bcl-2 pathway in the cochlea, indicating a possible role in ARHL progression [149].

#### 2.2.4. Sirtuins

The sirtuins (SIRT1-7) are a class of NAD^+^-dependent protein deacetylases that affect mitochondrial homeostasis, DNA damage repair, and aging [150,151]. Dysregulation of sirtuin activity is associated with numerous age-associated diseases, including neurodegeneration, insulin resistance, cardiovascular disease, and cancer [152]. Humans encode seven sirtuins (SIRT1-7) [153,154], localized to the following specific compartments: SIRT1, SIRT6, and SIRT7 in the nucleus [155], SIRT2 in the cytoplasm, and SIRT3, SIRT4, and SIRT5 in mitochondria [156]. SIRT1, the most well studied, may regulate lifespan by deacetylating [157] proteins such as p53, NF-κB, PGC-1α, and FOXO3a [156,158] and offers protection against aging-related diseases by promoting glucose homeostasis and insulin sensitivity [159]. SIRT2 reduces ROS production and mitochondrial dysfunction [160], while SIRT3 provides protection against oxidative stress and age-associated hearing loss [161]. Overexpression of SIRT2 [162] and SIRT6 in mice has been shown to improve longevity [163], in various mouse models.

The differential expression of sirtuin genes and proteins in the inner ear reflects their role in the aging process. Studies have shown that expression of SIRT1, 2, 4, 5, 6, and 7 increases whereas SIRT3 decreases in the vestibular organ, in the cochlea SIRT1, 3, and 5 decrease, with no changes observed in SIRT2, 4, 6, or 7 [164]. RNA-seq analysis has revealed altered sirtuin-related gene expressions in aging cochlear cells, including downregulation of *Sirt1* and *Sirt7*, and upregulation of *Sirt6* [35], suggesting a significant role for sirtuins in cochlear health and ARHL progression.

Additionally, there is a significant age-related decline in NAD^+^ levels, which is essential for sirtuin activity, due to the increased activity of NAD^+^-consuming enzymes like PARP-1 [165] and CD38 [166]. The loss of NAD^+^ impairs sirtuin activity and downregulates SIRT1 [167] and SIRT3 [168], leading to decreased deacetylation of p53, NF-κB, and IDH2 (isocitrate dehydrogenase 2). p53 acetylation triggers apoptosis, while acetylation of NF-κB induces a pro-inflammatory response [169], with both contributing to impaired auditory function. SIRT3 controls the deacetylation of IDH2 [161], which is crucial for maintaining mitochondrial function and protecting against oxidative stress in the cochlea [170,171]. Additionally, SIRT1 induces autophagosome formation by deacetylating ATG9A, a process impaired in aging hair cells of C57BL/6 mice, leading to impaired autophagy and accelerated ARHL progression [172].

#### 2.2.5. BubR1

The gene budding uninhibited by benzimidazole-related 1B (*Bub1B*) encodes the protein BubR1, a homolog of Mad3, a key regulator of the mitotic spindle assembly checkpoint (SAC) [173], which prevents chromatid mis-segregation and aneuploidy [173,174]. The gradual loss of BubR1 over time is thought to significantly contribute to aging [40], especially in the brain [175]. Hypomorphic BubR1 mice show increased senescence, early onset of aging-associated phenotypes, and a shortened lifespan [40]. Maintaining BubR1 levels has been shown to prolong lifespan and protect against premature aging markers, such as increased p16 and p19 expression in skeletal muscle and fat [40,110,162]. Restoring NAD^+^ or overexpression of SIRT2 can also increase BubR1 protein levels, suggesting a link between NAD^+^ decline and the loss of BubR1 activity during aging [40,162]. Mutations in BubR1 are associated with Mosaic Variegated Aneuploidy (MVA) syndrome, characterized by an increase in cells with aneuploidy, growth retardation, and facial dysmorphisms. These mutations lead to reduced functional BubR1 protein and a shortened lifespan [176], highlighting an important role for BubR1 in chromosomal stability and aging.

The decline of BubR1 with age leads to chromosomal instability, senescence, and the senescence-associated secretory phenotype (SASP), all of which contribute to the aging process, including the deterioration of auditory function [177]. BubR1 may influence hair cell function through multiple pathways, including its role as a pseudo-substrate inhibitor of APC/C^CDC20^ and its involvement in primary cilia formation, essential for cellular signal transduction [178]. Wnt/β-catenin signaling, critical for auditory system development [179], decreases in the auditory cortex of aged rats, along with reduced Bmi1 expression and increased p16, p19, and p53 levels, suggesting its involvement in ARHL. Overexpression of Bmi1, regulated by β-catenin, exerts anti-aging effects and confers resistance to apoptosis. Activation of Wnt/β-catenin signaling has been shown to increase Bmi1 expression, reduce apoptosis, and decrease neurodegeneration in the auditory cortex. Additionally, Wnt activation stabilizes Nrf2, a key regulator of oxidant metabolism, protecting against oxidative stress-induced hair cell damage [180,181]. The age-related decline in Nrf2 signaling may be linked to the degeneration of the auditory cortex and ARHL. These findings suggest potential avenues for future research into the role of BubR1 in ARHL.

#### 2.2.6. Key Aging Pathways and Their Role in ARHL

Overall, the primary mechanisms contributing to ARHL include mitochondrial dysfunction, which increases ROS production and leads to oxidative stress and apoptosis in cochlear cells. Additionally, the reduced levels of IGF-1 decrease the protection and maintenance of the cochlear hair cells, while the dysregulation of the mTOR and AMPK pathways further exacerbates oxidative damage and autophagy impairment. Furthermore, the decline in sirtuin activity and NAD^+^ levels with age impairs mitochondrial function and antioxidant defenses, contributing to cellular senescence and inflammation. The BubR1/Wnt/β-catenin signaling pathway also plays a role in regulating cellular senescence and apoptosis in the auditory system. These molecular disruptions collectively lead to the degeneration of hair cells and neurons in the auditory system, ultimately resulting in progressive hearing loss (Table 1).

## 3. Aging Interventions and Hearing Loss

**Figure 3:**
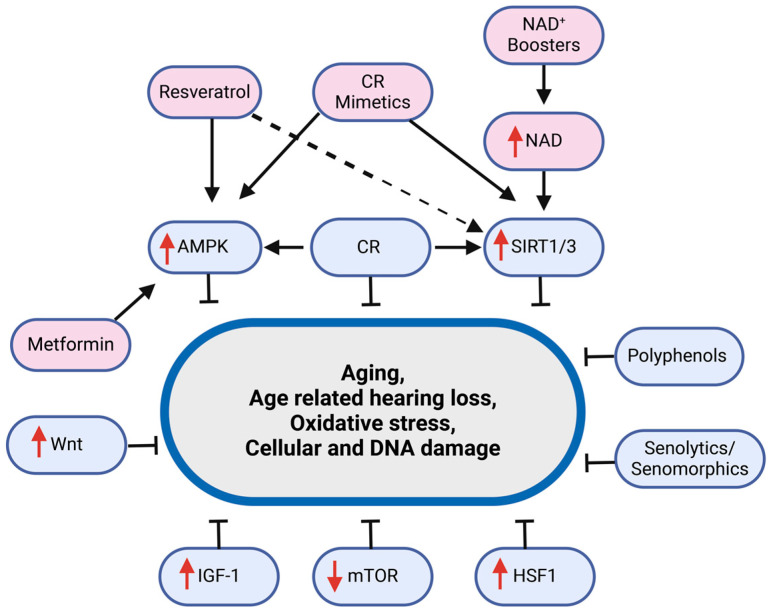
Model of key molecular targets for interventions in age-related hearing loss (ARHL). Caloric restriction (CR) and compounds like resveratrol activate AMPK, which can mitigate cellular damage. Increasing NAD^+^ levels, possibly through supplementation, supports the activation of SIRT1/3, which is associated with cellular longevity. Metformin, another AMPK activator, and interventions targeting Wnt, mTOR, and HSF1 pathways suggest potential avenues for treatment. A role for polyphenols and senolytics/senomorphics in managing age-related cellular changes is also shown.

As life expectancy has increased over the last century, the aging of the baby boomer generation, and the onset of their age-related diseases and conditions represent a significant financial burden for developed and developing countries. ARHL, as a prevalent condition affecting quality of life and cognitive function, is a particularly important target for intervention. The development of anti-aging interventions gives hope to postpone and protect against various chronic diseases, including ARHL, and improve the healthspan by establishing protective and rejuvenating responses through targeting age-related pathways, as illustrated in Figure 3 [182].

### 3.1. Rapamycin

Rapamycin has been found to extend lifespan in rodents [183,184] and delays age-related dysfunction in rodents and humans [185]. This compound functions by allosterically inhibiting mTOR, thereby influencing various cellular processes related to metabolism, proliferation, immune response, and cell survival [186]. Enhanced mTORC1 signaling within the cochlear neurosensory epithelium of aged mice has been linked to the progression of ARHL [140].

Peroxisomes in the inner ear hair cells have been found to regulate mTOR1 signaling. TSC1 and TSC2, located on peroxisomes in hair cells, are key negative regulators of mTORC1 and their loss increases mTORC1 activity, accelerating ARHL [140]. Rapamycin treatment has been observed to mitigate and delay age-related loss of cochlear basal outer hair cells in UM-HET3 mice [186] and Tsc1-cKO mice, by enhancing autophagy [140]. This enhancement of autophagy may help clear damaged cellular components and maintain hair cell function. Additionally, extended rapamycin treatment may activate Akt, further suggesting targeting mTOR as an approach for preventing ARHL [62].

However, it is crucial to consider that rapamycin also possesses immunosuppressive properties. This characteristic poses significant concern regarding its long-term use in aging populations, especially considering naturally declining immune functions [187,188]. This caveat emphasizes the following critical area of uncertainty: whether the immunosuppressive effects arise directly from mTOR regulation or are unique to rapamycin. This uncertainty suggests that alternative mTOR inhibitors might offer anti-aging benefits without compromising immune health.

### 3.2. Metformin

Metformin, approved for treating type 2 diabetes, is also recognized for its potential to delay aging and associated diseases [189]. Its lifespan-prolonging effects have been observed in model organisms, such as *C. elegans* and mice, through the activation of AMPK [190]. However, its effects appear dose-dependent and show variations across different species. For instance, the lifespan-extending impact of metformin is not consistently observed in all organisms, as seen in studies on *Drosophila* [191] and specific rat strains [192,193]. This inconsistency suggests a complex interaction of multiple factors, including dosage, species-specific responses, and environmental influences.

Metformin’s mechanism of action, involving increasing levels of AMP and subsequent activation of AMPK, mimics aspects of calorie restriction. This CR mimetic aspect contributes to its potential anti-aging effects, influencing autophagy, reducing inflammation, and improving cellular stress responses [194]. Furthermore, elevated ROS levels activate NF-κB, a key mediator of oxidative stress [195]. Antioxidant properties of metformin inhibit NF-κB gene expression, delaying the onset of aging-related symptoms [196]. In the auditory system, metformin has shown promise in animal models. It has been found to minimize the effects of noise-induced hearing loss in rats [197] and to significantly reduce neuronal apoptosis while delaying ARHL in mice treated with D-gal. This effect is achieved by regulating the unfolded protein response (UPR) via AMPK and ERK1/2 signaling pathways [198]. Moreover, metformin has been observed to inhibit neuroinflammation and reduce meningitis by protecting spiral ganglion neurons in the inner ear [199], which could contribute to maintaining auditory function and slowing the progression of ARHL.

### 3.3. NAD^+^ Boosters

Nicotinamide adenine dinucleotide (NAD^+^) is an essential cofactor for energy production, cell signaling, and the functioning of specific enzymes such as sirtuin deacetylases and poly-ADP-ribose polymerases [156,200]. In both human and animal models, aging is associated with a natural decline in NAD^+^ levels, leading to a reduction in the activity of these enzymes. Dietary choices, supplementation with precursor molecules, exercise, and the inhibition of enzymes that degrade NAD^+^, such as CD38, have been found to counter this decline, effectively restoring NAD^+^ levels in a majority of tissues [158].

Nicotinamide riboside (NR), a precursor of NAD^+^, has shown promise in enhancing the survival of outer hair cells in mouse models of Cockayne syndrome (CS), characterized by early-onset progressive sensorial hearing loss. Studies have reported correlation between aging, decreased NAD^+^ bioavailability, and SIRT1 expression in the mouse cochlea, and NAD^+^ supplementation has been shown to activate SIRT1 [200]. The NAD^+^/NADH ratio regulates sirtuins, PARP-1, and PGC-1α, which are important in cellular senescence [168]. NR supplementation has been demonstrated to protect the cochlea and synapses in mice from temporary and permanent threshold shifts by reducing oxidative damage to hair cells [201]. Similarly, long-term supplementation with NR restores NAD^+^ levels in the cochlea, improving high-frequency hearing and brain response in mice. This improvement occurs even after the onset of hearing loss [202]. NR also upregulates biological pathways associated with synaptic transmission and PPAR signaling in mouse models. Furthermore, NR influences lipid droplet pathways in the cochlea, inducing the expression of CIDEC and PLIN1 proteins downstream of PPAR signaling and important for lipid droplet growth [202]. The effects of NR appear independent of direct mitochondrial homeostasis, as mitochondrial RNA profiles remained unchanged, meaning that its beneficial effects on hearing loss might not be directly linked to mitochondrial functions. Therefore, these findings indicate that NR supplementation later in life can still be beneficial, even for mice already experiencing hearing loss at certain frequencies, and augmentation of NAD^+^ can be a sensible therapeutic option for ARHL [169]. In fact, increasing NAD^+^ levels may be similar to CR in combatting ARHL.

### 3.4. Resveratrol/Polyphenols

Polyphenols have attracted attention in the context of ARHL due to their antioxidant properties and effectiveness against oxidative-stress induced diseases. These compounds neutralize free radicals by inhibiting nitric oxide synthesis, decreasing lipid peroxidation, and enhancing cellular protection from apoptosis [203]. Resveratrol (trans-3,5,40-trihydroxystibene), a polyphenolic compound, has antioxidant potential [204] and also serves as an activator and natural agonist of SIRT1 [205].

The ability of resveratrol to inhibit apoptosis [205] has been linked to its role in activating AMPK in an SIRT1-dependent manner, leading to increased NAD^+^ levels and mitochondrial biogenesis and function [206]. In the organ of Corti of C57BL/6 mice, SIRT1 activation by resveratrol restored autophagy, thereby reducing hair cell death and preventing hearing loss [172]. Resveratrol has also demonstrated significant protective effects against auditory system damage by its ability to mitigate noise-induced hearing loss and preserve serotonin reuptake transporter (SERT) expression in C57BL/6J mice [207,208], as well as delay ARHL progression in C57BL/6 mice while reducing cochlear hair cell damage in male Fischer 344 rats [209]. Early treatment with resveratrol in male C57BL/6 mice has shown improvements in the hearing thresholds and reductions in the pro-apoptotic (Bax, Bak) and inflammatory (NF-κB, COX-2, iNOS) markers, with an increase in anti-apoptotic gene (Bcl-2, Bcl-xL) expression [210]. Moreover, long-term, low-dose resveratrol administration has been effective in reducing auditory threshold shifts and delaying the onset of ARHL by attenuating necroptosis in the aging cochleae [211]. It has also shown benefits in mitigating progressive peripheral neurodegeneration and hearing loss in a diabetic mouse model [212].

Recent studies have highlighted the potential of a combination of natural compounds to protect against ARHL. A mixture of 100 mg/kg polyphenols, including tannic acid and resveratrol, has improved auditory responses and oxidative stress markers in Sprague–Dawley rats [203,213]. The synergy of resveratrol and N-acetylcysteine (NAC) reduced ototoxic side effects of aminoglycosides in Wistar rats by increasing Gpx1 and Sod1 gene transcription and reducing inflammation [214]. Additionally, Ginkgo biloba extract (EGb761) has displayed antioxidant properties that prevent caspase-induced activities in cochleae of both young and aged mature Sprague–Dawley rats [215]. Curcumin effectively attenuates H_2_O_2_-induced apoptosis in auditory hair cells and prevents mitochondrial dysfunction through the activation of the Nrf2/HO-1 pathway via AKT in C57BL/6J mice [216]. These findings suggest that polyphenols may have potential in preventing ARHL. The diversity of research approaches in this area reflects the complexity of developing a universally applicable treatment. While resveratrol and other polyphenols show promise as protective agents against ARHL, further research is needed to standardize treatment protocols and fully understand their mechanisms across different biological systems.

### 3.5. Senolytics/Senomorphics

Senotherapeutics are drugs that target senescent cells, extend lifespan, and reduce the impact of aging and age-related diseases [217]. These drugs can be categorized as either senolytics or senomorphics [218]. Senolytics are designed to selectively induce apoptosis in senescent cells, mitigating tissue degeneration [217]. For instance, the senolytic cocktail of dasatinib, an anti-cancer agent, and quercetin, a flavonoid known to inhibit various tyrosine kinases [219], has been observed to effectively remove senescence cells and induce selective apoptosis [220]. Additionally, ATP-competitive HSP90 inhibitors, such as geldanamycin and 17-AAG, have shown senolytic activity both in cell culture and in vivo [221]. In addition, combining HSP90 inhibitors with drugs targeting anti-apoptotic pathways may extend the healthspan in humans [221].

Senomorphics, on the other hand, are designed to diminish the SASP, such as by decreasing the expression of pro-inflammatory cytokines [219]. Classes of senomorphics include inhibitors of NF-κB and JAK pathways [222]. Rapamycin exhibits a senomorphic effect, as it has been shown to decrease the SASP [223]. Another senomorphic approach is based on the interaction between p53 and Mouse Double Minute 2 (MDM2). This interaction leads to p53 ubiquitination and proteasomal degradation. MDM2 antagonists, Nutlin-3a and MI-63, increase p53 levels and reduce the SASP [219]. Furthermore, avenanthramice C (Avn C) activates AMPK and suppresses the NF-κB/p38 signaling pathway, thereby inhibiting the SASP in senescent human diploid fibroblasts [222]. However, the effectiveness of senotherapeutics in treating ARHL remains an open question. More research is needed to understand the role of senescence in ARHL and to determine the potential of senotherapeutics in mitigating this condition. Translating senotherapeutics from preclinical models to humans, particularly for ARHL, poses several challenges. Differences in cellular responses between animal models and humans, potential off-target effects, and the long-term impact on the auditory system require careful consideration. Clinical trials are essential to determine the safety, efficacy, and optimal use of these therapies in humans.

### 3.6. Calorie Restriction and CR Mimetics

Caloric restriction (CR), defined as a reduction in caloric intake without malnutrition [224], has been shown to delay aging and/or mitigate age-induced pathologies in both animal and cellular models [143]. CR may increase healthspan by downregulating key aging pathways, such as IIS and mTOR, while upregulating sirtuins and AMPK [225,226]. In *Saccharomyces cerevisiae*, CR benefits require *Sir2*, the human homolog of which is SIRT1 [227]. In *C. elegans*, mutations in *eat* genes mimic CR and extend lifespan, similar to *clk-1* gene mutations [228]. In mice, specifically female C3B10RF1 mice, reduced dietary intake has been shown to increase both mean and maximum lifespan [229,230]. CR improves mitochondrial function by promoting mitochondrial biogenesis and reducing ROS production, a process enhanced by SIRT1-mediated deacetylation of PGC-1α [231]. Moreover, CR reduces [232] the incidence of age-related diseases like neoplasia, sarcopenia [233], cardiovascular disease, and brain atrophy in rhesus monkeys [7]. Identifying targets of CR offers promising strategies for healthy aging, potentially complemented by PPAR agonists, due to their overlapping metabolic pathways [234].

CR appears to slow the progression of ARHL, although the exact molecular mechanisms still needs to be fully elucidated. A significant response to CR in the context of ARHL is the alteration of mitochondrial energy metabolism in auditory cells [168], reducing mtDNA damage and increasing energy metabolism, thereby decelerating aging in the auditory system [168]. Under CR, SIRT3 deacetylates and regulates IDH2, leading to IDH2-dependent NADPH production [235], which decreases oxidative stress and stimulates the antioxidant pathway in the inner ear [161], protecting against ARHL in mice on a calorie-restricted diet [235]. However, the absence of Sirt3 prevents the protective effect of CR against ARHL. In the cochlea specifically, CR inhibits p53-dependent apoptosis and downregulates pro-apoptotic proteins Bak and Bim [236].

The effects of diet and genotype on ARHL have been assessed by examining ABR thresholds and cochlear histopathology in various mouse strains (CBA/H-T6J, DBA/2J, C57BL/6J, BALB/cByJ, and WB/ReJ) on high-energy or calorie-restricted diets [237]. Genetic differences significantly influence ARHL outcomes in response to dietary interventions. Although calorie restriction (CR) extended the lifespan in some strains, it did not consistently affect cochlear pathology and, in some cases, even exacerbated it. Notably, CR effectively reduced cochlear pathology in B6 mice. These results show the need for further research into the genetic mechanisms at the cochlear level that influence the effectiveness of dietary interventions in ARHL, which could lead to more targeted and effective treatments based on genetic profiles [237]. These findings underscore the importance of considering genetic variability in ARHL research and suggest that future interventions may need to be tailored to individual genetic profiles for optimal effectiveness in preventing or treating ARHL.

While calorie restriction shows promise, its practical implementation can be challenging. This has led to the development of calorie restriction mimetics (CRMs). CRMs are designed to mimic the molecular, cellular, and physiological effects of CR, potentially offering a robust approach to combat aging-related diseases such as ARHL [238]. CRMs function by depleting AcCoA, inhibiting acetyltransferases and/or stimulating deacetylases, leading to protein deacetylation and the induction of autophagy [239]. Several compounds, including aspirin, hydroxycitric acid, resveratrol, and spermidine, have been characterized as CRMs [240]. Additionally, other compounds like NAD^+^ precursors, metformin, rapamycin, and curcumin, are considered potential CRMs due to the role of their molecular targets in CR [238,241]. PPAR agonists and ketone bodies also have the potential for CRMs [240].

It is important to note that, while CRMs typically target a single or a few pathways, aging and age-related diseases involve multi-functional pathways. Therefore, a more potent and synergistic effect may be achieved by combining multiple CRMs or pairing a CRM with other health-promoting compounds, such as antioxidants [238]. This multifaceted approach could enhance the efficacy of CRMs in mitigating the complexities of aging and related conditions, including ARHL.

Preclinical therapies targeting ARHL focus on key mechanisms involving mTOR inhibition, AMPK activation, NAD^+^ boosting, polyphenols, and calorie restriction. These interventions, along with senotherapeutics and caloric restriction mimetics (CRMs), show promise in combating aging and age-related diseases, including ARHL (Table 2). Ongoing clinical trials, though not directly addressing ARHL, may provide valuable insights into these interventions’ potential for age-related conditions. For instance, nicotinamide riboside supplementation is being studied for its effects on cognitive function and brain metabolism in older adults (NCT03482167). Multiple studies are evaluating the potential for metformin in delaying age-related diseases and extending a healthy lifespan (NCT02432287), and investigations into the effects of resveratrol on cognitive function in older adults are ongoing (NCT01126229). While these trials do not directly address ARHL, their outcomes could offer valuable insights into the potential of these interventions for age-related conditions, including hearing loss.

## 4. Conclusions

ARHL is a prevalent chronic auditory disorder affecting one-third of the global population. This geriatric disease often results in social isolation, communication barriers, depression, and dementia, presenting a major socioeconomic challenge in both developed and developing countries. The pathophysiology of ARHL involves various components of the auditory system, including sensory hair cells, stria vascularis, afferent spiral ganglion neurons, and the central auditory pathways. Identifying the connection between aging and ARHL at the molecular level will clarify the molecular mechanism of ARHL, including pathways such as senescence, AMPK, mTOR, insulin/IGF-1, and sirtuins. However, further studies are required to elucidate the direct relationship between these aging-related pathways and ARHL. Understanding these molecular mechanisms and recognizing the influence of CR on ARHL could provide a way for innovative therapeutic strategies. Currently, there are no effective treatments for ARHL, which underlines the urgent need to explore and identify novel molecular targets for this condition. The development of small molecule regulators targeting these pathways holds great promise for the prevention and treatment of ARHL, addressing a critical gap in geriatric healthcare.

## Figures and Tables

**Table 1:** Key aging pathways and their role in age-related hearing loss (ARHL).

Pathway	Role in Aging	Effects on Hearing	References
IGF-1 Signaling	Decreases with age	- Protects cochlear hair cells - Promotes regeneration - Inhibits apoptosis - Reduced levels lead to hearing loss	[128,129,130,131,132,133]
mTORC1 Signaling	Hyperactivated in aged cochlear cells	- Increases oxidative stress - Impedes autophagy - Contributes to hair cell damage and hearing loss	[139,140]
AMPK Pathway	Activated by ROS; inhibited in AMPKα1 depleted mice	- Promotes autophagy under stress - Excessive activation linked to hearing loss - Inhibition may reduce apoptosis and protect hearing	[148,149]
Sirtuin Pathway	SIRT1, SIRT3, SIRT5 decrease with age in cochlea; NAD^+^ levels decline	- Regulates mitochondrial function - Influences autophagy and antioxidant defence - Decreased levels lead to oxidative stress and hearing loss	[35,164,168,169,170,171,172]
BubR1/Wnt/β-Catenin Signalling	Decreased BubR1, Wnt/β-catenin signalling in the aged auditory system, involves CDC20, p16, p19, p53	- Regulates cellular senescence - Influences chromosomal stability and apoptosis - Decreased signalling contributes to hair cell degeneration and hearing loss	[179,180,181]
ROS and Mitochondrial Dysfunction	Increased ROS and decreased antioxidant enzymes with age	- Promotes oxidative stress, apoptosis, and senescence in cochlear cells - Leads to progressive hearing loss	[61,62,63,64,65,66,67,68]
Mitophagy and Autophagy	Impaired mitophagy and autophagy with age	- Essential for cellular homeostasis and hair cell survival - Impairment leads to senescence and exacerbates hearing loss	[90,91,92,93,94]
Cellular Senescence	Accumulation of senescent cells with age; regulated by mTOR and AMPK pathways	- Contributes to ARHL through oxidative stress and inflammation - ROS-induced DNA damage triggers p53 signaling, promoting senescence - Impaired mitophagy leads to increased cellular senescence	[42,62,98,102,103,104,105,111,113,114]

**Table 2:** Aging interventions and their impact on ARHL.

Intervention	Mechanism of Action	Effect on ARHL	Benefits	Limitations/Considerations	Research Stage	References
Rapamycin	mTOR inhibition, enhances autophagy	Delays ARHL by reducing mTORC1 activity in cochlear cells	Protects cochlear hair cells; Enhances autophagy	Potential immunosuppressive effects: Alternative mTOR inhibitors may be needed	In vivo (mouse models); Clinical trials for other conditions	[140,186]
Metformin	AMPK activation, mimics calorie restriction	Reduces inflammation, delays ARHL, reduces neuronal apoptosis	Well-established safety profile; Multiple beneficial effects	Dose-dependent effects; Not consistently effective across all species	In vivo (mouse models); Clinically approved (for diabetes)	[197,198,199]
NAD^+^ Boosters (e.g., NR, NMN)	Restores NAD^+^ levels, activates sirtuins	Protects cochlea, enhances survival of hair cells, reduces oxidative stress	Can protect against hearing loss even in advanced age; Enhances mitochondrial function	Optimal dosage and long-term effects still under investigation	In vivo (mouse models); Early-stage clinical trials	[200,201,202]
Resveratrol/ Polyphenols	SIRT1 activation, reduces oxidative stress, enhances autophagy	Delays ARHL, protects against cochlear damage and neuronal loss	Natural compounds with multiple health benefits; Acts as a SIRT1 agonist	Bioavailability issues; Optimal dosage unclear	In vivo (mouse models); Clinical trials for other conditions	[172,207,208,209,210,211,212,213,214,215,216]
Senolytics (e.g., Dasatinib + Quercetin)	Induces apoptosis in senescent cells, reduces SASP	Potential to mitigate tissue degeneration and extend healthspan	Targets fundamental aging processes; Potential for periodic treatment	More research needed for ARHL-specific effects; Optimal timing and dosing unclear	Preclinical research; Early clinical trials for other conditions	[217,218,219,220,221,222,223]
Calorie Restriction and CR Mimetics	Activates autophagy, modulates mitochondrial energy metabolism, promotes protein deacetylation	Slows ARHL progression, reduces oxidative damage in mitochondria, potential to delay ARHL by targeting aging-related pathways	Comprehensive health benefits beyond hearing; No drug side effects; May provide CR benefits without dietary restriction; Multiple targets	Challenging to implement long-term; Effectiveness may vary with genetics; Specific effects on ARHL need more research; Optimal compounds still under investigation	In vivo (mouse models); Limited human studies; Preclinical research; Some compounds in early clinical trials	[235,236,237,238,239,240,241]
